# Cepharanthine hydrochloride: a novel ferroptosis-inducing agent for prostate cancer treatment

**DOI:** 10.3389/fphar.2025.1536375

**Published:** 2025-02-24

**Authors:** Jing-Song Guan, Jing Jia, Ze-Xiu Huang, Yu-Qing Zhou, Jing-Jie Zheng, Qi-Man Lin, Yi-Fei Wang, Jiang-Lin Fan, Yao Wang

**Affiliations:** ^1^ Guangdong Provincial Key Laboratory of Large Animal Models for Biomedicine, South China Institute of Large Animal Models for Biomedicine, School of Pharmacy and Food Engineering, Wuyi University, Jiangmen, China; ^2^ College of Life Science and Technology, Jinan University, Guangzhou, Guangdong, China; ^3^ International Healthcare Innovation Institute (Jiangmen), Jiangmen, Guangdong, China

**Keywords:** cepharanthine, ferroptosis, androgen receptor, prostate cancer, combination therapy

## Abstract

**Background:**

Ferroptosis is an intracellular iron-dependent cell death that is distinct from apoptosis, necrosis, and autophagy. Increasing evidence indicated that ferroptosis plays a crucial role in suppressing tumors, thus providing new opportunities for cancer therapy. The drug cepharanthine, commonly used to treat leukopenia, has been discovered to function as an anticancer agent to multiple types of cancer via diverse mechanisms. However, the effect of cepharanthine on prostate cancer remains unclear.

**Methods:**

A semi-synthetic derivative of cepharanthine, cepharanthine hydrochloride (CH), is used in this study due to its better water solubility and bioavailability. The prostate cancer cell lines LNCaP, 22Rv1, PC3 and xenograft mouse models are used for detecting the anti-tumor effect of CH *in vitro* and *in vivo*. Types of cell death including ferroptosis are detected by flow cytometry using annexin V and total/lipid reactive oxygen species probes, drug combination of CH with ferroptosis inhibitor/ion chelator, and the appearance of mitochondria under a transmission electron microscopy. The mechanism is investigated by high-throughput transcriptome analysis and transcription factor function analysis of androgen receptor.

**Results:**

CH inhibits cellular functions and trigger ferroptosis in prostate cancer cells. Mechanistic research revealed both common and distinct pharmacological mechanisms of CH-induced ferroptosis in different prostate cancer cells. High-throughput transcriptome analysis revealed that ferroptosis-related genes are significantly regulated in androgen receptor-dependent cells 22Rv1 and LNCaP, and less significantly in androgen receptor-independent cell PC3. Furthermore, CH was found to reduce the gene expressions and protein levels of GPX4 and FSP1 through modulating the activity of the androgen receptor signaling pathway, but not through its transcription factor activity. In addition, CH upregulated ACSL4 and downregulated DHODH, with the combined regulatory outcomes synergistically inducing ferroptosis. An *in vivo* experiment employing CH and ion chelator-treated nude mice validated the mechanism by which CH induces ferroptosis to combat prostate cancer.

**Conclusion:**

This study has identified CH as a novel ferroptosis-inducing agent for the treatment of prostate cancer. The multiple mechanisms we found provides strong evidence for the eventual clinical application of the drug.

## Background

Ferroptosis is a type of regulated cell death that relies on iron and is activated when cellular membranes accumulate toxic levels of lipid peroxides (Dixon et al., 2012). This phenomenon has gained significant attention in the field of cancer research due to its distinct mechanisms and appearance compared to other forms of cell death, such as apoptosis. Therefore, ferroptosis holds great potential for cancer therapy (Lei et al., 2022). Targeting ferroptosis may provide new therapeutic opportunities for refractory cancers.

Progress has been made in understanding the role of ferroptosis in tumor biology and therapy. Cancer-associated signaling pathways govern ferroptosis in cancer cells (Hassannia et al., 2019). Tumor suppressors such as p53 and BAP1 establish ferroptosis as a barrier to cancer development (Jiang et al., 2015; Zhang et al., 2018a), while evasion of ferroptosis contributes to tumor progression and resistance (Angeli et al., 2019). Some cancer cells are intrinsically susceptible to ferroptosis due to their distinctive metabolism, high reactive oxygen species (ROS) levels, and specific mutations (Zou et al., 2020; Wu et al., 2019; Viswanathan et al., 2017). Disrupting ferroptosis defense systems could be fatal to these cancer cells while sparing normal cells. Ferroptosis is also triggered by various cancer therapies, making it a targetable vulnerability (Lei et al., 2020; Guo et al., 2018). Ferroptosis inducers have great potential in cancer therapy (Liang et al., 2019). Targeting the key enzymes and molecules involved in the ferroptosis process is one potential approach to developing ferroptosis inducers for cancer treatment.

Although the mechanism and metabolic pathway of ferroptosis are still poorly understood, studies on inducers and inhibitors have made new developments with the emergence of new ferroptosis targets (Du and Guo, 2022). Glutathione peroxidase 4 (GPX4) and ferroptosis suppressor protein 1 (FSP1) constitute two major ferroptosis defense systems (Yang et al., 2014; Doll et al., 2019). Acyl-CoA synthetase long chain family member 4 (ACSL4) is involved in the synthesis of endogenous PUFAs, turning arachidonic acid into acyl-CoA, which reacts further to produce phospholipid hydroperoxides (Doll et al., 2017). Dihydroorotate dehydrogenase (DHODH) is the rate-limiting enzyme in *de novo* pyrimidine nucleotide biosynthesis and complements mitochondrial GPX4 in reducing peroxidized membrane phospholipids. DHODH inhibitors enhance ferroptosis (Amos et al., 2023). GPX4 activity depends on glutathione produced from the activation of the cystine-glutamate antiporter SLC7A11 (Chen et al., 2021). Ferritin is composed of ferritin light chain (FTL) and ferritin heavy chain 1 (FTH1). Inhibiting the expression of iron response element binding protein 2 (IREB2), the main transcription factor of iron metabolism, can significantly increase the expression of FTL and FTH1, thereby inhibiting ferroptosis induced by erastin (Gammella et al., 2015). Transferrin receptor (TFRC) is considered as a biomarker of ferroptosis in cell cultures or tissues (Gao et al., 2015). SAT1 is a transcriptional target of P53 and an important rate-limiting enzyme for polyamine catabolism. Activation of SAT1 induces lipid peroxidation and ferroptosis induced by ROS (Ou et al., 2016). HMOX1 is an important biomarker, the pro-ferroptotic effect of which has been verified experimentally in human aortic smooth muscle cells (Wu et al., 2022). It can also increase the susceptibility of the human lens epithelium to erastin (Liao et al., 2023).

Autophagy plays an important role in regulating ferroptosis through the control of cellular iron homeostasis and the generation of ROS. Autophagy can promote ferroptosis in cancer cells by degrading and recycling iron-storage proteins, such as ferritin, which can release iron and promote lipid peroxidation and oxidative stress (Gao et al., 2016). In addition, a study found that inducing autophagy in cancer cells increased the sensitivity of these cells to ferroptosis-inducing agents, such as erastin and RSL3 (Zhou et al., 2019). Conversely, autophagy can impede ferroptosis in cancer cells by eliminating damaged or oxidized lipids that can initiate ferroptosis (Zhang et al., 2018b). Therefore, the impact of autophagy on ferroptosis in cancer cells is complex and context dependent.

As the mechanisms of ferroptosis in cancer are gradually better understood, ferroptosis inducers have become indispensable in cancer therapy. We aim to discover new ferroptosis inducers from natural product drugs to accelerate the process of clinical application. In our previous study of hepatocellular carcinoma (Wang et al., 2020), Cepharanthine hydrochloride (CH), a semi-synthetic derivative of Cepharanthine, was found to induce mitophagy, which is a specific form of autophagy that involves the selective degradation of damaged or dysfunctional mitochondria. Furthermore, from our preliminary experiments, CH induced non-apoptotic cell death in prostate cancer (PCa). Therefore, we hypothesize that CH may have a ferroptosis-inducing effect. Cepharanthine is used in Japan for the treatment of leukopenia after radiotherapy, as well as alopecia areata and alopecia totalis. In this study, we demonstrate that CH induces ferroptosis in multiple ways in PCa cells. These findings also provide new therapeutic options for PCa, especially for apoptosis-resistant cells.

## Materials and methods

### The cells and the reagents

LNCaP, 22Rv1, PC3, and DU145 cells were purchased from the American Type Culture Collection (ATCC). PC-3M cells were obtained from the Institute of Cell Research, Chinese Academy of Sciences (Shanghai, China). The cell lines were all authenticated by short tandem repeat (STR) test. LNCaP, 22Rv1, DU145, and PC-3M cells were maintained in RPMI-1640 medium (SH30809.01, Hyclone, Utah, United States) supplemented with 10% fetal bovine serum (FBS). PC3 cells were cultured in Ham’s F12-K medium (21127022, ThermoFisher, Waltham, MA, United States) with 10% FBS.

Cepharanthine hydrochloride (CH), a semi-synthetic derivative of cepharanthine (A1443), was purchased from MUST BioTechnology (Chengdu, China). Deferoxamine (DFO, S5742), ferrostatin-1 (Fer-1, S7243), RSL-3 (S8155), and wortmannin (S2758) were purchased from Selleck Chemicals (Houston, TX, United States). Matrigel (354234) was obtained from Corning Costar (Lowell, United States). Testosterone (T1500) and methyl-thiazolyldiphenyl-tetrazolium bromide (MTT, M2128) were purchased from Sigma-Aldrich (St. Louis, MO, United States). Cell Counting Kit-8 (CCK-8, K1018) was obtained from APExBIO (Houston, Texas, United States). TRIzol reagent (15596–026) and the BCA protein assay kit (23227) were purchased from Invitrogen (Waltham, MA, United States). PrimeScript RT reagent kit (RR047A) and SYBR Premix DimerEraser (RR091A) were purchased from TaKaRa (Kusatsu, Shiga, Japan). Enhanced chemiluminescence (ECL) blotting detection kit (34580) was purchased from ThermoFisher Scientific (Waltham, MA, United States). Bovine serum albumin (BSA, 1027–106) was from Gibco (Waltham, MA, United States). RIPA buffer (P0013C) was purchased from Beyotime (Shanghai, China). Crystal violet (DC079) was from Genview (Beijing, China). Paraformaldehyde (BL539A) was from Biosharp (Hefei, China).

### Animal studies

All animal work was performed in accordance with the Institutional Animal Care and Use Committee of Wuyi University (No. N2022013) and AAALAC guidelines. Maximum tumor volume should not exceed 1000 mm^3^ for mice. 22Rv1 xenograft models were established by subcutaneous (s.c.) injection of 5 × 10^6^ cells in Matrigel into the left flank of male nude mice when the animals were 6–7 weeks of age. When tumors reached between 100–200 mm^3^, as measured by a caliper using the formula V = 1/2 ab^2^ (a, long axis; b, short axis), the mice were randomized into four groups of five mice each. The grouped mice were then subjected to different treatments intraperitoneally (i.p.): saline (Model), 100 mg/kg DFO, 30 mg/kg CH, and a combination of 100 mg/kg DFO with 30 mg/kg CH. The sizes of the tumors were measured every other day. All the animals were sacrificed on the 13th day. Livers of the mice were collected for hepatotoxicity study.

### Cell viability assay

Cells were plated in 96-well plates at a density of 4,000 cells per well and allowed to adhere overnight. Subsequently, the cells were subjected to incremental concentrations of CH treatment for either 24 or 48 h. Following the treatment, MTT solution (0.5 mg/mL) was added to the cell culture medium and incubated with the cells for an additional 4 h at 37°C. The supernatant was subsequently aspirated, and 100 μL of DMSO was added. The optical density of the dissolved formazan was quantified at 570 nm using a multifunctional microplate detector (Synergy Neo2, BioTek, Winooski, VT, United States). The optical density (OD570) of the control cells was designated as 100% viability.

### Colony formation assay

The cells were seeded in 6-well plates at a density of 1 × 10^3^ cells per dish and allowed to grow for 7–9 days. When colonies containing 20–30 cells were formed, they were treated with different concentrations of CH for an additional 9 days. The colonies were finally fixed with 4% paraformaldehyde and stained with 0.1% crystal violet.

### Invasion assay

The transwell system (3422, Corning Costar, Lowell, United States) was used for invasion experiments. Matrigel (0.5 mg/mL) was added to the upper chamber, and a suspension of 2 × 10^5^ cells in 100 μL serum-free medium was inoculated after the matrigel solidified. In the bottom chamber, 500 μL of medium containing 20% FBS and CH was added. Following 24 h of culturing, the invaded cells were stained with 0.1% crystal violet, and images of the invaded cells were captured using an invert microscope (RVL-100-G, ECHO, San Diego, CA, United States).

### Scratch wound healing assay

The confluent cells were scratched in a line across the bottom of the culture dish with a pipet tip. The cells were then cultured with a medium containing 2% FBS. Cell migration was observed at 24 h and 48 h, respectively. The micrographs show the extent of scratch closure achieved under normal conditions in comparison to conditions inhibited by the addition of CH. Cell migration quantification was evaluated by measuring the average distance of the central gap.

### Flow cytometry analysis

Cells were treated with CH for 24 h. For apoptosis analysis, the cells were harvested and stained with AnnexinV Alexa Fluor 488/PI reagent (FXP022-050, 4A Biotech Co., Beijing, China). For total reactive oxygen species (ROS) analysis, the cells were stained using a Total ROS Assay Kit (88–5930, Invitrogen, CA, United States). For lipid peroxidation detection, we used BODIPY (581/591) C11 (D3861, Invitrogen, CA, United States) as the probe. This probe incorporates into membranes where it undergoes a fluorescence emission shift upon peroxidation. The stained cells were then detected by a flow cytometer (CytoFLEX LX, Beckman Coulter, Indianapolis, IN, United States).

### Transfection

Transfections were performed using polyethylenimine (PEI) for Western blotting analysis. For siRNA transfection, non-targeting control siRNA and siRNAs specific for *human AR* were constructed by Sangon Biotech (Shanghai, China). The sequences of the siRNAs (5′-3′) are: siRNA#1 (+), CCU​GCU​AAU​CAA​GUC​ACA​CAU​TT; siRNA#2 (+), CGC​GAC​UAC​UAC​AAC​UUU​CCA​TT; siRNA#3 (+), GAA​AUG​UUA​UGA​AGC​AGG​GAU​TT.

### Western blotting assay

Cells were lysed in RIPA buffer containing Tris (pH 7.4, 50 mM), NaCl (150 mM), 1% NP40, 0.5% sodium deoxycholate, 0.1% SDS, and protease inhibitor. The concentrations of the extracted proteins were determined via BCA assay. The lysates were mixed with a loading buffer and denatured by heating at 95°C for 10 min. Protein lysates with an equal total protein content were loaded onto an SDS-PAGE (8%–12%) gel and run under constant voltage to separate proteins. The proteins on the gel were then transferred to PVDF membranes (ISEQ00010, Millipore, Billerica, MA, United States) and probed with the indicated primary antibodies overnight and subsequent species-specific HRP-conjugated secondary antibodies. The immunoreactive bands were detected using an ECL blotting detection kit. The band intensity was quantified by ImageJ software.

Primary antibodies of AR (5153), LC3B (2775), and GAPDH (2118) were purchased from Cell Signaling Technology (Bost on, MA, United States). PARP (sc-8007), cleaved-PARP (sc-56196), Bcl-2 (sc-509), Caspase-3 (sc-56053), P62 (sc-28359), ATG5 (sc-133158), FSP1 (sc-377120), GPX4 (sc-166570), and AR (441) (sc-7305) were obtained from Santa Cruz Biotechnology (Dallas, TX, United States). DHODH (14877-1-AP) was from Proteintech (Manchester, United Kingdom). ACSL4 (ab155282) was from Abcam (Cambridge, United Kingdom).

### Transmission electron microscopy assay

LNCaP cells at a confluence of 60%–70% were treated with CH for 12 h and fixed in 2.5% glutaric dialdehyde at 4°C overnight. After fixation, dehydration, infiltration, and embedding, the samples were sliced into ultra-thin sections and stained with uranyl/lead citrate. Images were captured under a transmission electron microscope (TEM for short, HT7700, Hitachi, Tokyo, Japan).

### Real-time quantitative polymerase chain reaction (q-PCR)

Total mRNA was extracted using the TRIzol reagent. The quantity and quality of RNA were determined using a spectrophotometer (DS-11 FX+, DeNovix, Wilmington, United States), and then the RNA was reversely transcribed into cDNA using a PrimeScript™ RT reagent kit with gDNA eraser (Perfect Real Time; RR047A, TAKARA, Kyoto, Japan) according to the manufacturer’s instructions. The q-PCR was performed using a 2 × SYBR Green qPCR Master Mix (Low ROX; B21703, Selleck, Texas, United States) in the real time fluorescence quantitative PCR instrument (LightCycler 96, Roche, Basel, Switzerland) in a 20 μL reaction. The primers are shown in Table 1.

**TABLE 1:** Primers of the genes.

Gene name	Forword (5′-3′)	Reverse (5′-3′)
GPX4	ACC GAA GTA AAC TAC ACT CAG	GGC GAA CTC TTT GAT CTC TT
ANDROGEN RECEPTOR	GGT GAG CAG AGT GCC CTA TC	GAA GAC CTT GCA GCT TCC AC
FSP1 (AIFM2)	AGT AGT GGG GAT AGA CCT GAA GA	CCA CCA CGA TGA ACC GTG A
ACSL4	GCT ATC TCC TCA GAC ACA CCG A	AGG TGC TCC AAC TCT GCC AGT A
DHODH	GGC GTG GAG ACA CCT GAA AAA G	CAG GTG TTC AGC ATA GAA ACG C
FTH1	TGA AGC TGC AGA ACC AAC GAG G	GCA CAC TCC ATT GCA TTC AGC C
STEAP3	ATC TTT GTG GCT GTG TTC CG	TTG CTC TGT AGG GTT GCT CA
TFRC	AAG ACA GCG CTC AAA ACT CG	TGC AGC CTT ACT ATA CGC CA
SLC7A11	TCT CCA AAG GAG GTT ACC TGC	AGA CTC CCC TCA GTA AAG TGA C
SAT1	CGG AAT CCC AGC TCC ACT TA	TAA AGG GCC TAC GGA CTT GG
HMOX1	GGT CAT CCC CTA CAC ACC AG	CAG ACA GGT CAC CCA GGT AG
GAPDH	GAG TCA ACG GAT TTG GTC GT	GAC AAG CTT CCC GTT CTC AG

### RNA-Seq transcriptome analysis

High-throughput sequencing was performed using the TRIZOL method (T9424, Merck, Rahway, NJ, United States) to prepare of RNA samples. The quality and integrity of the processed samples were assessed by measuring nucleic acid concentration with a Nanodrop2000 (Thermo Fisher, Waltham, MA, United States) and evaluating integrity using an Agilent 2100 or LabChip GX (PerkinElmer, Waltham, MA, United States). Sequencing was conducted with the DNBSEQ-T7RS High-throughput Sequencing Kit (FCL PE150) V3.0 (940–000266–00, BGI Genomics, Shenzhen, China). The final library was loaded at a concentration of 1 pmol, as determined by a Qubit 3.0 fluorometer (Thermo Fisher, Waltham, MA, United States) with the Qubit dsDNA HS Assay Kit (1000017571, BGI Genomics, Shenzhen, China). Differentially expressed genes (DEGs) were identified with P values adjusted using the Benjamini–Hochberg method to control the false discovery rate. A corrected P value threshold of 0.05 and a minimum absolute fold change of 2 were applied to define significant differential expression. Additionally, KOBAS software was used to assess the statistical enrichment of DEGs in KEGG pathways, providing insights into their potential biological roles and interactions. The transcriptome data generated in the present study can be found in the NCBI BioProject under accession number PRJNA1157790.

### Statistical analysis

All data are shown as mean ± SD from at least 3 independent experiments. The 2-tailed Student's t-test and one-way ANOVA were used for the statistical analysis. Statistical significance was defined as *, P < 0.05; **, P < 0.01; ***, P < 0.001.

## Results

### CH inhibits functions of the PCa cells

**FIGURE 1:**
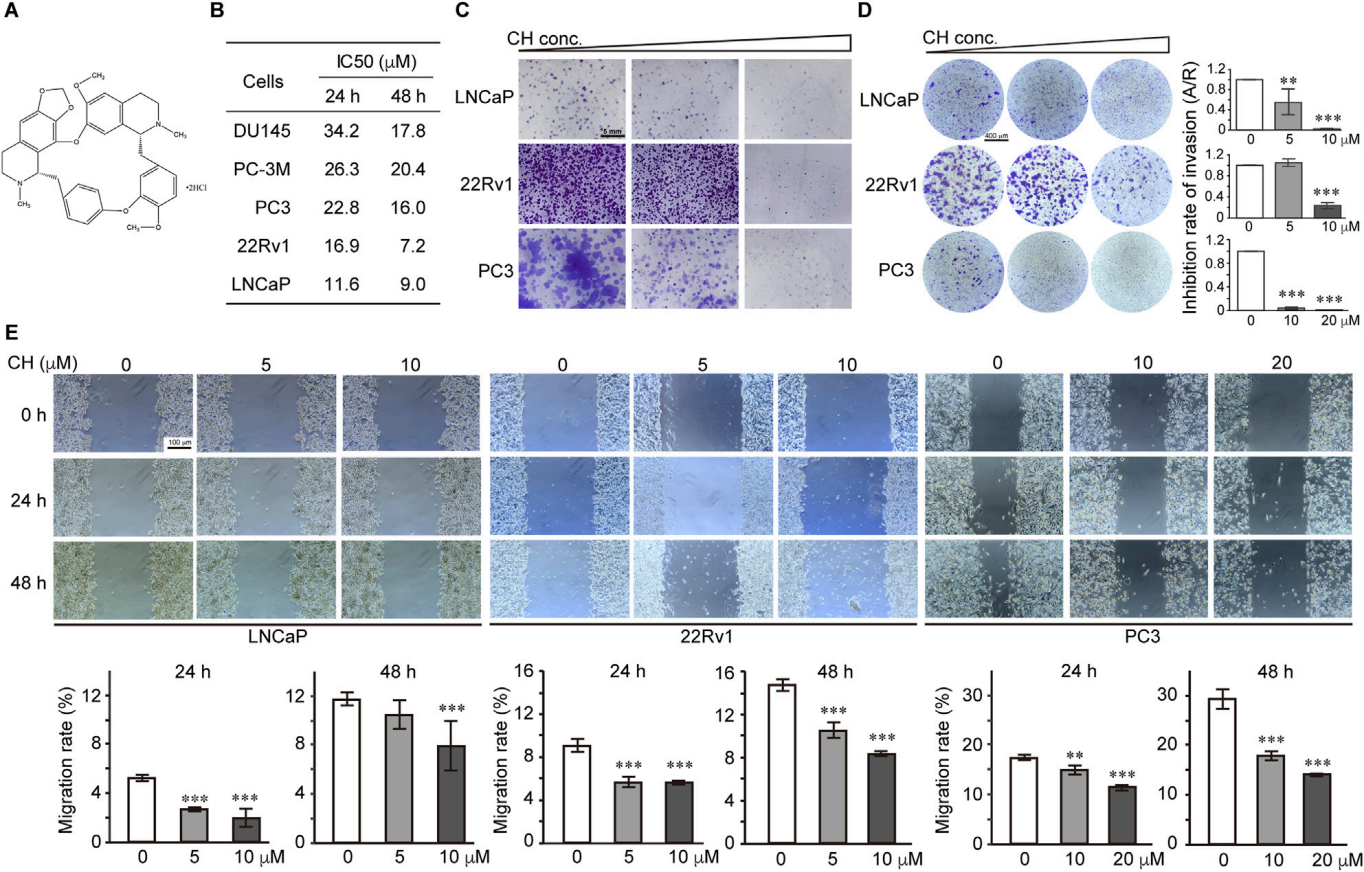
Effect of CH on PCa cell functions. **(A)** The chemical structure of CH. **(B)** The cytotoxicity assays of CH in different PCa cells. IC50 values at 24 h and 48 h are shown. **(C)** Colony formation assays. CH concentrations used in this assay are: LNCaP, 0, 5, 10 μM; 22Rv1, 0, 5, 10 μM; PC3, 0, 10, 20 μM. **(D)** Transwell invasion assay. All the cells were treated with CH for 48 h. CH concentrations used in this assay are: LNCaP, 0, 5, 10 μM; 22Rv1, 0, 5, 10 μM; PC3, 0, 10, 20 μM. A/R, area ratio. **(E)** Cell migration examined by scratch assay. Cells were scratched and treated with CH for 24 h or 48 h. Migration distances were measured and the migration rate (%) were quantified. **, *p* < 0.01; ***, *p* < 0.001 compared with DMSO treated cells.

The structure of CH used in this study is shown in Figure 1A. To evaluate the inhibitory effect of CH on PCa cells, we performed a cell viability assay using AR-independent cells DU145, PC-3M, PC3, and AR-dependent cells 22Rv1 and LNCaP. From the IC50 values shown in Figure 1B, we found that CH exhibits cytotoxicity across various PCa cell types, among which 22Rv1 and LNCaP cells showed the highest sensitivity. We primarily used these 2 cell lines for the following experiments. Since LNCaP and 22Rv1 are both androgen receptor (AR)-dependent prostate cancer cells, we also selected the AR-independent cell line PC3 for comparative studies in our subsequent experiments, as PC3 requires less drug concentration to achieve similar effects, making it more drug-sensitive. CH is shown here to be a promising drug to inhibit PCa *in vitro*, due to its inhibitory effects on colony formation (Figure 1C), transwell invasion (Figure 1D), and scratch wound healing (Figure 1E) in these three PCa cells.

### CH induces ferroptosis in PCa cells

**FIGURE 2:**
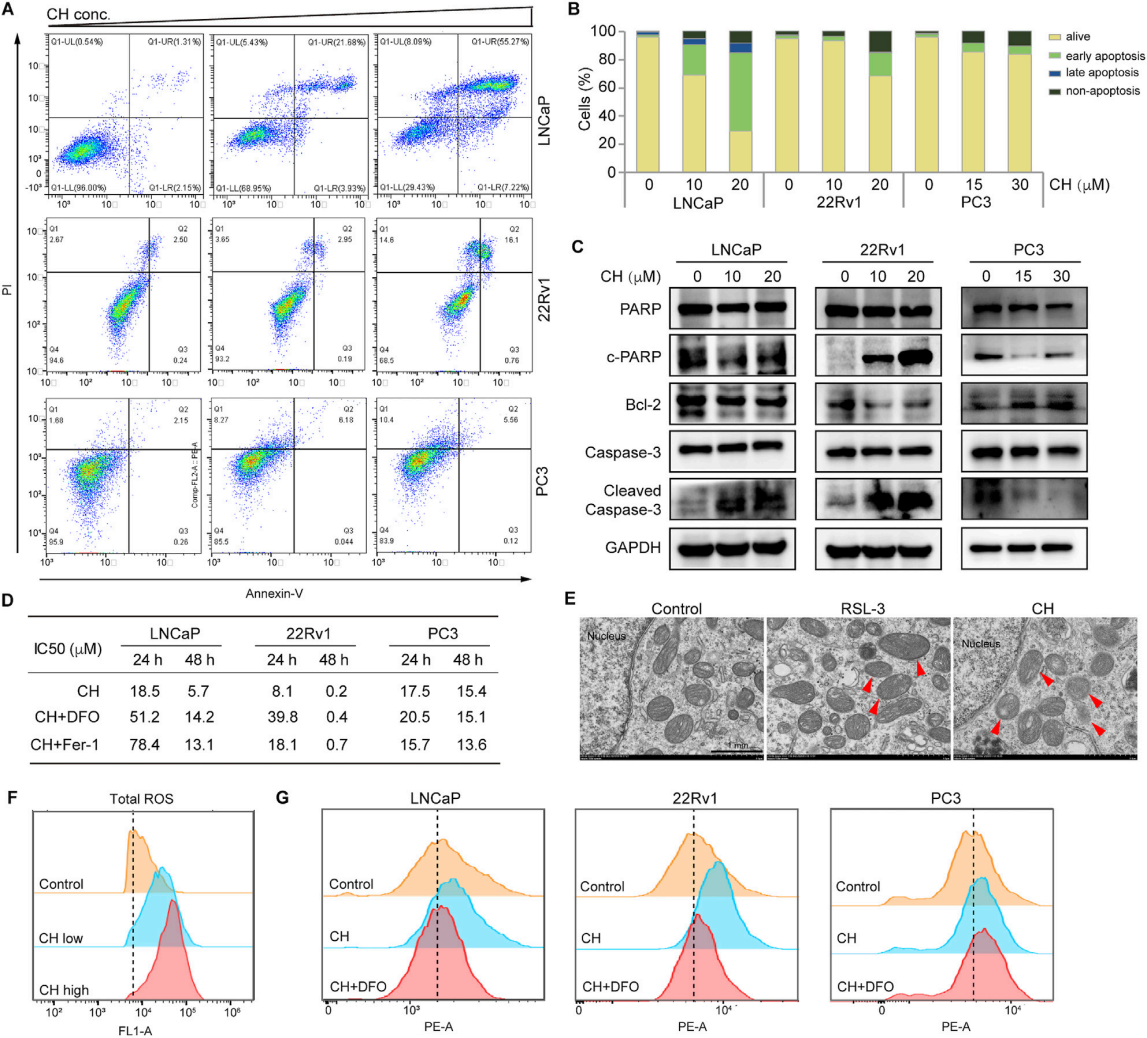
CH induced ferroptosis in PCa cells. **(A)** Cell apoptosis detected via flow cytometry analysis. Cells were treated by CH for 24 h and CH doses in this assay are: LNCaP, 0, 10, 20 μM; 22Rv1, 0, 10, 20 μM; PC3, 0, 15, 30 μM. **(B)** Quantitative analysis of apoptotic cells in A. **(C)** Apoptosis-related protein levels in PCa cells. **(D)** Cell viability assays. LNCaP: DFO, 5 μM; Fer-1, 60 nM. 22Rv1: DFO, 3 μM; Fer-1, 3 μM. PC3: DFO, 3 μM; Fer-1, 3 μM. **(E)** Electron microscopy of LNCaP cells. Arrow heads indicate mitochondria. Scale bar, 1 μm. **(F)** Total ROS analyzed by flow cytometry in LNCaP cells. **(G)** Lipid peroxidation analyzed by flow cytometry. DFO concentrations for LNCaP, 22Rv1 and PC3 are: 60 nM, 3 μM, 3 μM. CH concentrations for LNCaP, 22Rv1 and PC3 are: 20 μM, 20 μM, 30 μM.

To identify the nature of cell death induced by CH, we investigated the apoptotic pathway firstly. CH can induce apoptosis in LNCaP and 22Rv1 cells, but simultaneously, it can also trigger a certain degree of non-apoptotic cell death (Figures 2A, B). However, for PC3 cells, non-apoptotic cell death is the main form of cell death under the action of CH. Similarly, apoptotic marker analysis via Western blotting confirmed that LNCaP and 22Rv1 cells underwent a certain degree of apoptosis, while PC3 cells should have undergone a different form of cell death (Figure 2C). To determine the specifics of CH-induced non-apoptotic cell death, we further examined the ferroptosis pathway, as our previous studies found that CH can induce autophagy in various cells (Wang et al., 2019; Wang et al., 2020). As autophagy is also associated with ferroptosis, we speculate that the agent may induce ferroptosis in PCa cells. Iron chelator DFO and ferroptosis inhibitor Fer-1 were subsequently used in cell viability assays to validate the existence of CH-induced ferroptosis (Figure 2D). It is noteworthy that the concurrent administration of DFO or Fer-1 markedly diminished the susceptibility of LNCaP and 22Rv1 cell lines to CH, thereby highlighting the ferroptosis inducing potential of CH in these 2 cells. Under a TEM, the mitochondria of LNCaP cells treated by CH underwent shrinkage and increased membrane density compared to the mitochondrial features induced by RSL-3 (Kinowaki et al., 2021), a well-known ferroptosis inducer (Figure 2E). The total ROS levels of LNCaP cells (Figure 2F) and lipid peroxidation detection of LNCaP and 22Rv1 cells (Figure 2G) confirmed that CH induced ferroptosis in these AR-dependent cells. For PC3 cells, an AR-independent cell, the levels of CH-induced lipid peroxides did not significantly increase, and iron chelator also did not have a protective effect. These findings suggest that CH can trigger both apoptosis and ferroptosis in PCa cells, with a particularly strong effect observed in AR-dependent cells.

### CH regulates autophagy

**FIGURE 3:**
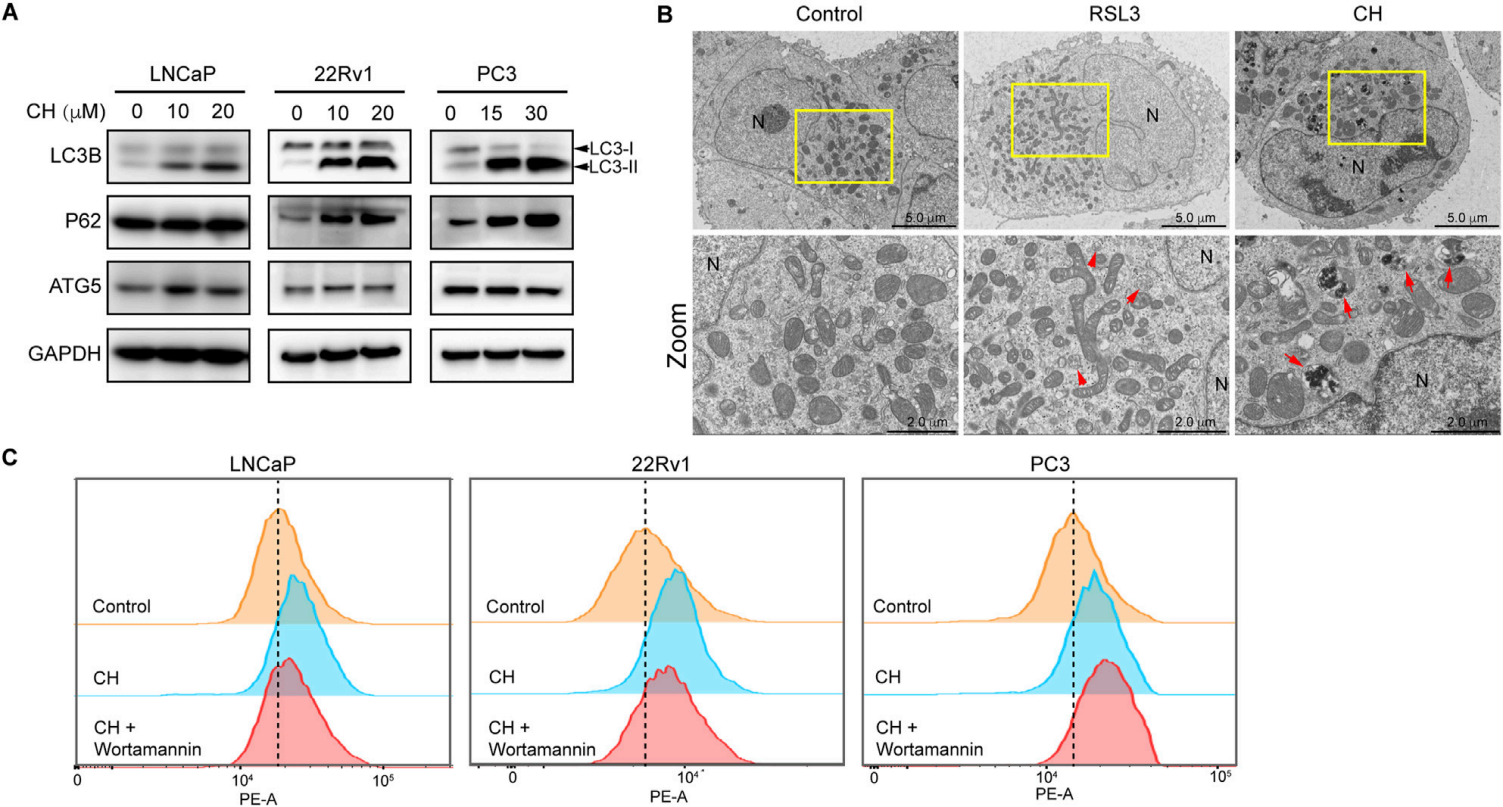
CH regulates autophagy. **(A)** Autophagy-related protein levels in CH treated PCa cells. **(B)** Electron microscopy of LNCaP cells. RSL3, 10 ng/mL; CH, 20 μM; N, nucleus; Arrows indicate autophagosomes. **(C)** Lipid peroxidation analyzed by flow cytometry. CH concentrations for LNCaP, 22Rv1 and PC3 are: 20 μM, 20 μM, 30 μM. Wortmannin, 10 μM.

There is a complex relationship between ferroptosis and autophagy. To clarify the regulatory relationship between ferroptosis and autophagy under the treatment of CH, and to determine whether CH-induced ferroptosis is related to autophagy, we first investigated the autophagy flux in all 3 cell lines. As shown in Figure 3A, protein levels of LC3-II and the ratio of LC3-II/LC3-I were increased in a dose-dependent manner in CH-treated cells, indicating CH initiated autophagy in all 3 cell lines. The protein sequestosome 1 (p62/SQSTM1) is an autophagy receptor and a selective substrate for autophagy. P62 accumulation observed here indicates a reduced autophagic flux. Autophagic cell death may be the main reason for the toxicity of CH to PC3 cells. TEM analysis revealed that autophagosomes appeared frequently in RSL3 treatment, as indicated by red arrows in Figure 3B (middle). In the cells treated with CH, numerous autophagosomes can also be observed, but these autophagosomes may have engulfed more organelles, such as mitochondria (Figure 3B, right). We next employed the autophagy inhibitor wortmannin in flow cytometry assay (Figure 3C). Wortmannin can restore CH-induced lipid peroxidation elevation in LNCaP and 22Rv1 cells, but not in PC3 cells. overall, we illustrated that CH can induce incomplete autophagic flux in PCa cells, preventing autophagic substrates from being recycled by the cells, which may be the main reason for the toxicity of CH to PC3 cells. Blocking autophagy can reduce lipid peroxides caused by CH, indicating that CH-induced accumulation of autophagic cargos promotes ferroptosis.

### Transcriptome analysis of CH-induced ferroptosis

**FIGURE 4:**
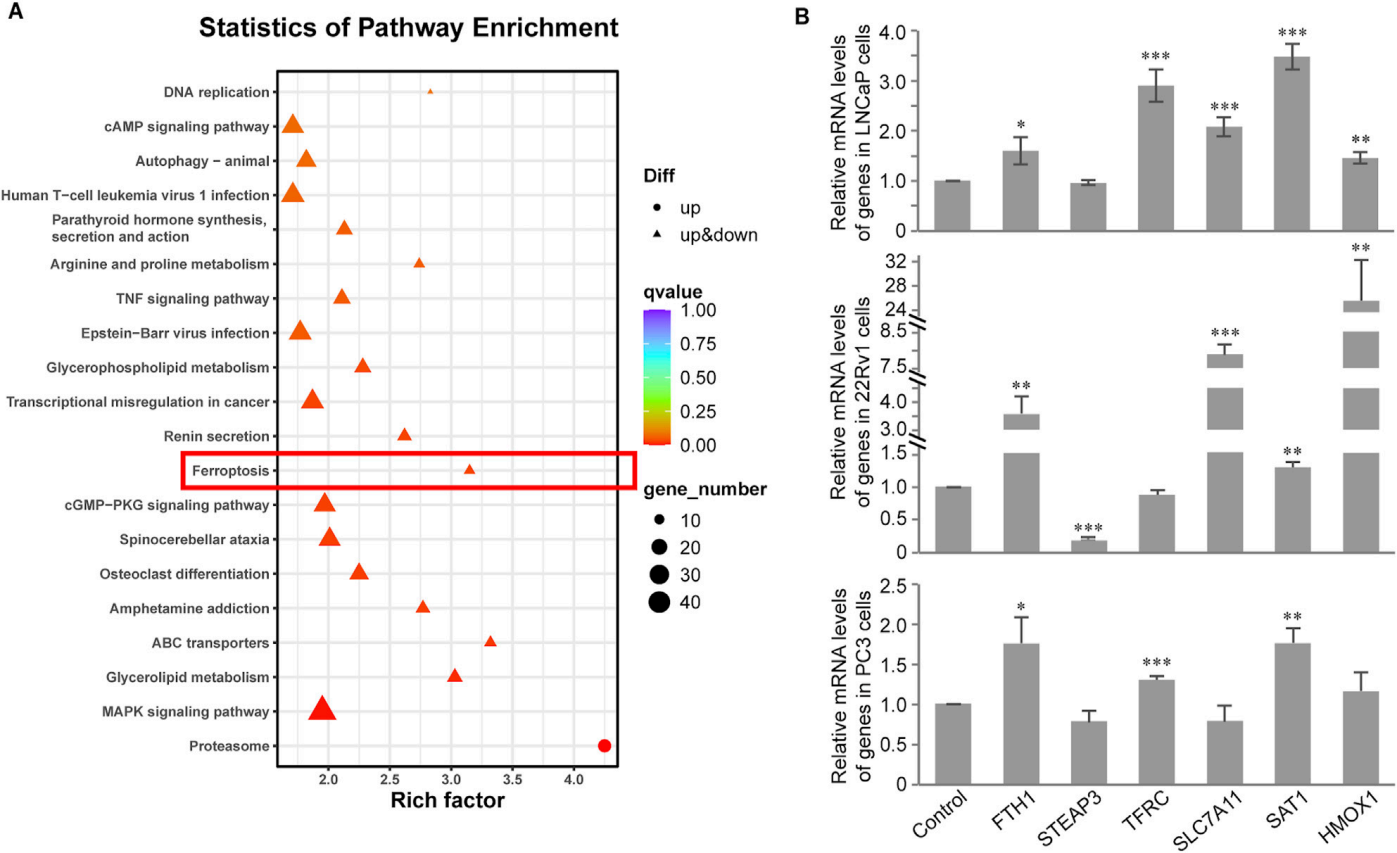
Transcriptome analysis of CH treated PCa cells. **(A)** KEGG pathway enrichment analysis in LNCaP cells. **(B)** Validation of ferroptosis associated genes by real-time PCR assay in PCa cells. *, *p* < 0.05; **, *p* < 0.01; ***, *p* < 0.001 compared with DMSO treated cells.

To systemically study the mechanisms of CH-induced ferroptosis, we performed transcriptome analysis and pathway enrichment analysis of DEGs using the KEGG database (KEGG, 2022). From the results of 22Rv1 cells, the significance of DEGs in the ferroptosis pathway ranks 12th (Figure 4A), much higher than LNCaP and PC3 cells. Next, we performed quantitative real-time PCR validation experiments on these ferroptosis pathway-related genes that exhibited significant regulation in the 22Rv1 cell line, across the three different PCa cell lines (Figure 4B). In 22Rv1 cells (Figure 4B, middle), FTH1, SLC7A11, and HMOX1 were increased more than twofold. Among the three upregulated genes, FTH1 and SLC7A11 were both negative regulators in ferroptosis. Note that STEAP3 is downregulated by CH significantly, which results in ferroptosis induction. From the above findings, the mechanism of ferroptosis induced by CH in 22Rv1 cells is complex. The regulation of ferroptosis-related genes by CH does not solely target the induction of ferroptosis, but rather represents a comprehensive outcome of multi-gene regulation. In LNCaP cells (Figure 4B, top), there are only three genes—TFRC, SLC7A11, and SAT1—regulated more than twofold by CH treatment. However, in PC3 cells (Figure 4B, bottom), no gene was significantly regulated more than twofold. Overall, the mechanism of CH-induced ferroptosis in PCa varies among cells.

### CH regulates GPX4 and FSP1 via nuclear AR

**FIGURE 5:**
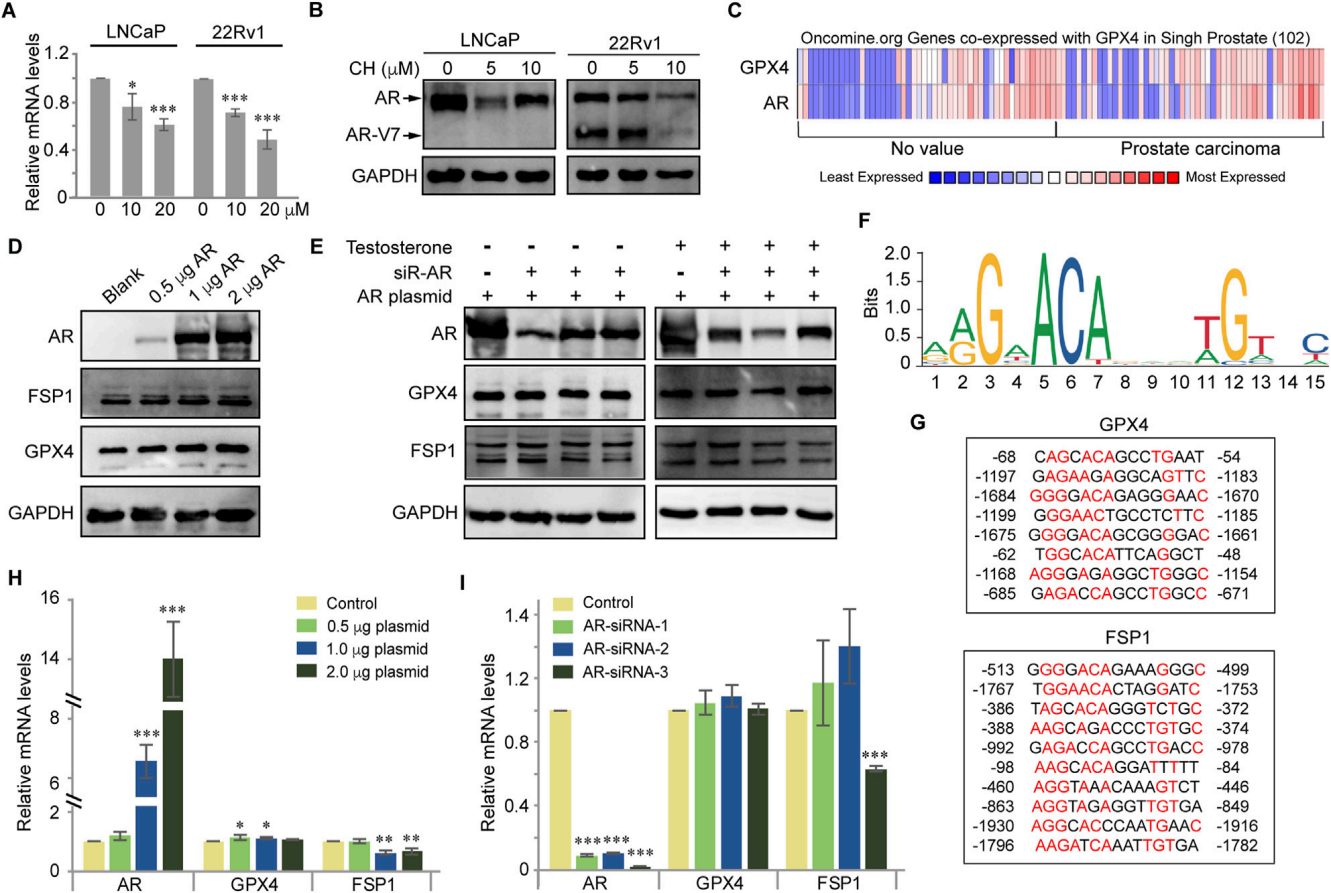
CH regulates GPX4 and FSP1 in PCa cells. **(A)** Quantitative detection of GPX4 by real-time PCR. **(B)** Protein levels of AR and AR variants in LNCaP and 22Rv1 cells. **(C)** Coexpression of GPX4 and AR in cancer microarray database and integrated data-mining platform (Oncomine). **(D)** Protein levels of GPX4 and FSP1 in exogenous AR expressed HEK293T cells. Testosterone, 10 nM. **(E)** Effect of AR knockdown on protein levels of GPX4 and FSP1 in HEK293T cells. Testosterone, 10 nM. AR plasmid, 2 μg. **(F)** JASPAR database predicted the sequence logo of *homo sapiens* AR. **(G)** JASPAR database predicted the binding sites of AR to the promotor regions of GPX4 and FSP1. **(H)** Relative mRNA levels of GPX4 and FSP1 in exogenous AR expressed HEK293T cells. Testosterone, 10 nM. **(I)** Effect of AR knockdown on relative mRNA levels of GPX4 and FSP1 in HEK293T cells. Testosterone, 10 nM *, *p* < 0.05; ***, *p* < 0.001 compared with DMSO treated cells.

FSP1 is a glutathione-independent ferroptosis inhibitor molecule, which exerts a ferroptosis inhibition role parallel to GPX4. Therefore, we aim to determine the effect of CH on these two ferroptosis defense factors. As the above experiments have shown that under the treatment of CH, PC3 cells hardly exhibit ferroptosis, our subsequent mechanistic studies focused on LNCaP and 22Rv1 cells. Firstly, we found that CH downregulated the mRNA levels of GPX4 in LNCaP and 22Rv1 cells (Figure 5A). Meanwhile, CH reduces AR protein and its truncated variant AR-V7 (Figure 5B). To elucidate the intrinsic relationship between AR and GPX4, we browsed Oncomine (ONCOMINE, 2020) (the website has now ceased its operation) to determine AR and GPX4 expression, and found co-expression of these two genes within a research of PCa containing 102 cases (Figure 5C). As AR functions both as a nuclear receptor and a transcription factor, our initial validation focuses on the nuclear translocation of AR and its regulatory effects on GPX4 and its parallel ferroptosis suppressor FSP1. In AR-overexpressing cells treated with the AR ligand testosterone, the expression of protein AR leads to a modest increase in the protein levels of GPX4 and FSP1 (Figure 5D). However, in cells overexpressing protein AR and subsequently knocked down by siRNAs, in the presence of androgen, the expression of both GPX4 and FSP1 proteins decreases with the reduction of AR. However, in the absence of androgen, the levels of protein GPX4 and FSP1 are not influenced by AR levels (Figure 5E). This indicated that only nuclear localized AR exerts slight regulatory effects on both proteins. Furthermore, to verify whether GPX4 and FSP1 serve as target genes of AR, acting as a transcription factor, we used JASPAR (JASPAR, 2022) to predict whether AR could bind to the promoters of the two genes. The sequence logo representing conserved DNA binding sites where AR binds is shown in Figure 5F. Intriguingly, JASPAR predicts that AR is likely the promoter of both GPX4 and FSP1, because there are numerous potential binding domains in the promoter regions of these two genes (Figure 5G). However, regardless of AR overexpression or knockdown, the transcription levels of genes GPX4 and FSP1 show no significant and consistent accompanying changes (Figures 5H, I). Therefore, we infer that AR is not a direct transcription factor for genes GPX4 and FSP1. Overall, we conclude here that CH moderately regulates the expressions of GPX4 and FSP1 via nuclear AR, but does not involve its transcription factor function.

### CH regulates ACSL4 and DHODH

**FIGURE 6:**
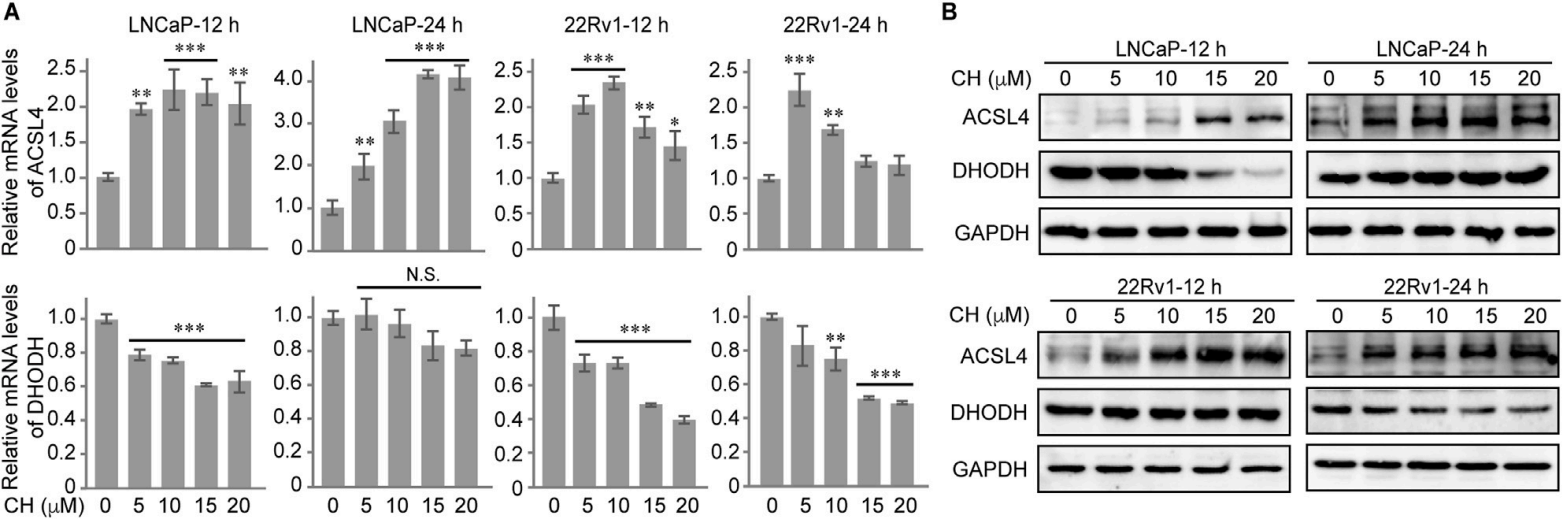
CH regulates ACSL4 and DHODH in PCa cells. **(A)** Relative mRNA levels of ACSL4 and DHODH in CH treated PCa cells. **(B)** Protein levels of ACSL4 and DHODH in CH treated PCa cells. *, *p* < 0.05; **, *p* < 0.01; ***, *p* < 0.001 compared with DMSO treated cells.

In our subsequent research, we conducted exploratory studies on other important regulatory factors of ferroptosis. Our findings revealed that the regulation of ACSL4 and DHODH by CH played a pivotal role in this process. CH up-regulated the transcriptional levels (Figure 6A, top) and protein levels of ACSL4 (Figure 6B, top), but down-regulated the transcriptional levels (Figure 6A, bottom) and protein levels (Figure 6B, bottom) of DHODH in LNCaP cells and 22Rv1 cells. The regulation of CH on ACSL4 and DHODH synergistically promotes the occurrence of ferroptosis.

### CH induced ferroptosis *in vivo*

**FIGURE 7:**
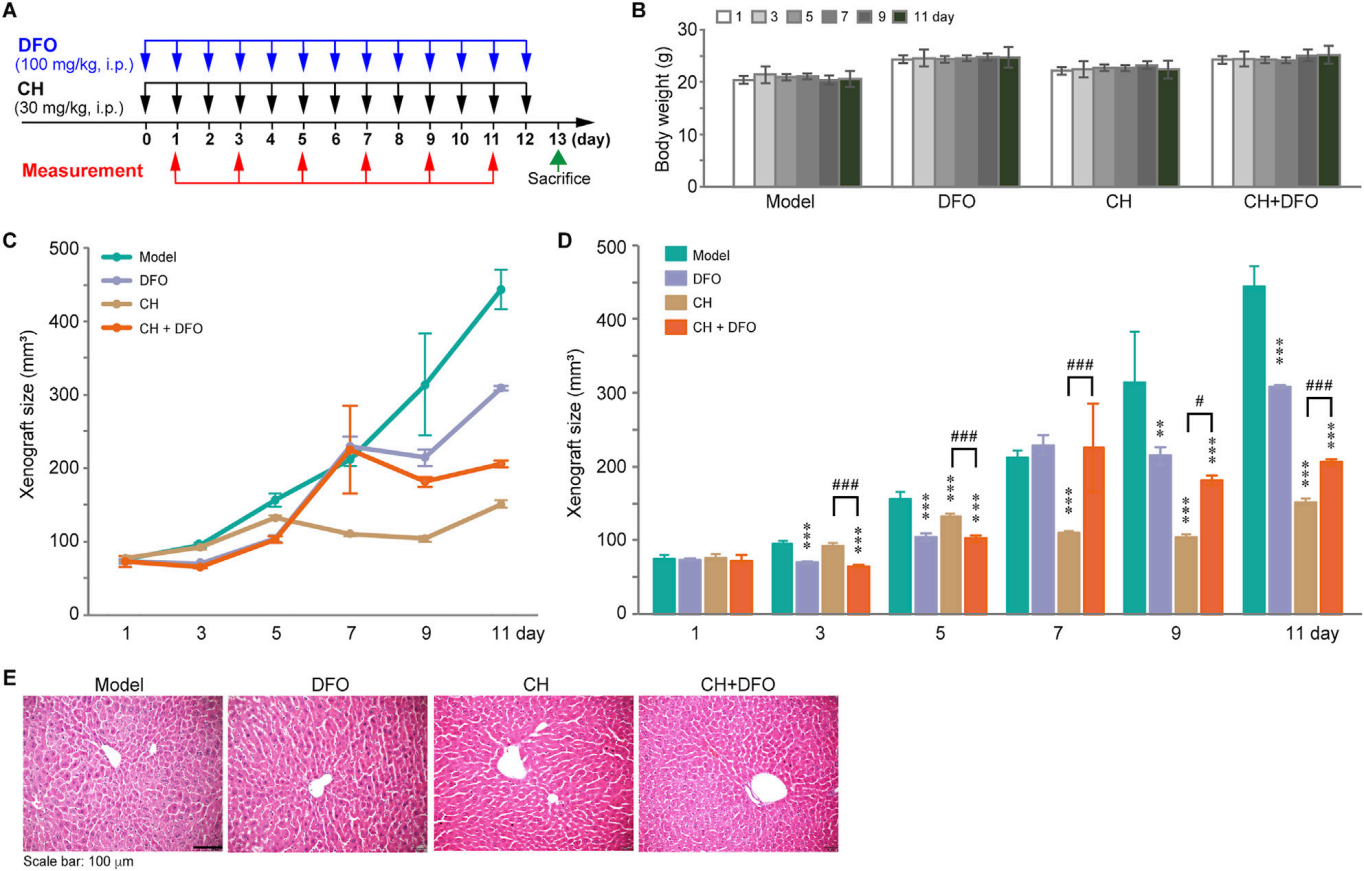
The anticancer effect of CH via ferroptosis in animal models. **(A)** Design and dosing of *in vivo* experiment. **(B)** Body weights of the mice. **(C)** Change of the xenograft size over time. **(D)** Statistics of xenograft size. **, *p* < 0.01; ***, *p* < 0.001 compared with model group. ^#^, *p* < 0.05; ^###^, *p* < 0.001. **(E)** HE stainings of the livers.

To investigate the effect of CH on inducing ferroptosis *in vivo*, a xenograft mouse model was established. The 6–7-week-old nude mice were treated with CH (30 mg/kg) or CH combined with DFO (100 mg/kg) intraperitoneally for 12 days (Figure 7A). The animals were sacrificed on the 13th day. The body weights and tumor sizes were measured every other day. Figure 7B shows that during the administration period, no significant body weight change was observed within each group. Interestingly, a xenograft growth curve revealed a significant inhibition of tumor growth in the CH group. However, when combined with DFO, the xenografts exhibit a reduced sensitivity to CH (Figure 7C). To better illustrate the differences among the groups, we statistically analyzed tumor size and presented the results in the form of a bar chart (Figure 7D). From the chart, we found that DFO itself had some anti-tumor effects throughout the therapy period except the seventh day, while CH exhibited anti-tumor effect from the fifth day onward. Data from the seventh day to the 11th day showed significant differences in tumor size between the CH group and CH + DFO group, indicating the critical role of ferroptosis in the pharmacological mechanism of CH. HE stainings of the liver tissue showed no obvious hepatotoxicity in both groups during the 12-day experiment period (Figure 7E).

## Discussion

As a leucocyte-stimulating agent, Cepharanthine has been previously explored for the treatment of various cancers, excluding PCa (Wang et al., 2023). In the current study, we identified the anti-PCa effect of CH, and further revealed the mechanism by which CH induces ferroptosis. Mechanically, transcriptome analysis revealed changes in multiple ferroptosis-related factors induced by CH, especially in AR-dependent PCa cells. In addition, the drug synergistically regulated key regulators of ferroptosis, including GPX4, FSP1, ASCL4, and DHODH. Specifically, nuclear AR protein—but not its transcriptional activity—participated in the regulation of GPX4 and FSP1. Animal experiments confirmed the effects of CH inducing ferroptosis in combating PCa, as discovered *in vitro*. Therefore, we infer that CH is a novel applicable ferroptosis inducer in AR-dependent PCa cells.

CH was shown here to trigger mixed types of cell death (including apoptosis, autophagy, and ferroptosis) in PCa cells. Likewise, other natural product like gallic acid (GA) was reported, which activates iron-dependent cell death mechanisms with apoptotic, ferroptotic, and necroptotic features (Tang and Cheung, 2019) For natural products with complex structures and multiple functional groups, such as CH and GA, it is not difficult to understand that they can induce multiple cell death pathways in the same cell. However, few relevant reports exist, and researchers often overlook other cell death pathways when they discover a new one in pharmacological research. Due to the complexity of the *in vivo* environment and cell composition, the effects of a drug can vary at different concentrations and in different tissues. A comprehensive and integrated evaluation of drug-induced cell death is crucial for understanding the role of drugs in a given disease model.

Despite increasing evidence, the role and pathophysiology of autophagy during ferroptosis remain unclear. It is known that ferroptosis is a type of cell death that is regulated by autophagy, which controls iron levels and ROS production. Ferritin stores iron in a less harmful form, but under certain conditions, autophagy promotes ferritin degradation, leading to the release of toxic iron and oxidative stress, ultimately causing ferroptosis (Gao et al., 2016). On the other hand, knockout or knockdown of Atg5 can suppress ferroptosis by reducing intracellular iron levels, highlighting the importance of autophagy in this process (Hou et al., 2016). In this study, we found that CH-induced autophagy occurred in all 3 cell lines, although the autophagic flux was not intact, as indicated by the aggregation of P62. Lipid peroxidation flow cytometry analysis of LNCaP and 22Rv1 cells suggested that autophagy inhibition could partially alleviate the increased CH-induced lipid peroxidation levels, indicating the critical role of autophagy in CH-induced ferroptosis. This effect was not observed in PC3 cells, where the increase in lipid peroxidation levels induced by CH was not significant, and autophagy inhibitors could not reverse this mild change. The regulation relationship between autophagy and ferroptosis varies depending on the cell and tissue, and it cannot be assumed that inducing autophagy will necessarily lead to ferroptosis. Although Cepharanthine has been found to regulate autophagy in multiple tumors (Gao et al., 2017; Wang et al., 2020; Li et al., 2022), whether the drug can induce ferroptosis in these tumors must still be separately verified.

To explore the mechanism of CH-induced ferroptosis, we first conducted transcriptomic analysis on three PCa cell lines and found that the drug-induced ferroptosis signal was strongest in 22Rv1 cells. Therefore, we validated these ferroptosis-related genes in all 3 cell lines via quantitative PCR and found that these genes were indeed most affected in 22Rv1 cells, followed by LNCaP cells and PC3 cells. This phenomenon was consistent with the results of flow cytometry and cell viability experiments using ferroptosis inhibitors, which showed that CH-induced ferroptosis is significant in LNCaP and 22Rv1 cells, but not in PC3 cells. Therefore, we hypothesized that the occurrence of ferroptosis may be related to AR function, which we later confirmed in experiments. AR nuclear translocation indeed affected the expression of GPX4 and FSP1, but the specific mechanism of this effect requires further investigation. In addition, based on the transcriptome results, it is evident that CH consistently regulates FTH1 and SAT1 across three different cell lines. It remains to be determined whether these two genes are target genes of CH. Interestingly, in LNCaP and 22Rv1 cells, CH consistently affects the expression of SLC7A11 and HMOX1. Further investigation is needed to determine whether these two genes are functionally related to the AR transcription factor.

We observed significant variability in the IC50 values at the 48-h time point between the cytotoxicity experiments presented in Figures 1B, 2D. This discrepancy is primarily attributed to a combination of technical and biological factors inherent to prolonged drug exposure assays. Specifically, the inconsistency in cell passage numbers between the two experiments likely contributed to the observed differences, since the passage number can influence cellular sensitivity to drugs. Additionally, subtle variations in microenvironmental conditions, such as differences in serum lots or minor pH shifts during the extended 48-h incubation, may modulate drug sensitivity. Moreover, the transition from the MTT assay (Figure 1B) to the CCK8 assay (Figure 2D) introduced methodological variability. The MTT assay relies on mitochondrial dehydrogenase activity to reduce tetrazolium salts into insoluble formazan crystals, which require solubilization steps that can introduce variability. In contrast, the CCK8 assay utilizes water-soluble tetrazolium salts that are directly reduced by cellular dehydrogenases, avoiding the need for crystal dissolution. These differences in assay mechanisms may lead to variations in signal stability and sensitivity, particularly at longer incubation times such as 48 h. Despite these technical considerations, the relative potency rankings and mechanistic conclusions drawn from our experiments remain robust and consistent with our overall findings.

In our animal experiments, we also found that the iron chelator DFO alone has a certain anti-tumor effect. Even before ferroptosis was discovered and named, the anti-cancer activity of iron chelators has been found in different experimental contexts including cell experiments, animal experiments, and clinical trials (Buss et al., 2003). In addition to their individual effectiveness, iron chelators can also enhance the efficacy of other anti-cancer treatments through synergistic effects. According to our research findings, the co-administration of DFO and CH not only failed to exhibit a synergistic anti-tumor effect, but also reduced the tumor’s sensitivity to CH. This directly confirms that CH exerts its anti-PCa effects by inducing ferroptosis.

In conclusion, our research has found that CH, as a natural product drug-derived compound, possesses a biological function of inducing ferroptosis in PCa through multiple mechanisms. However, whether the drug can induce ferroptosis in other tumors still needs to be explored, as whether a given cancer is more sensitive or resistant to ferroptosis induction is dictated by its specific genetic background.

## Data Availability

The datasets presented in this study can be found in online repositories. The names of the repository/repositories and accession number(s) can be found below: https://www.ncbi.nlm.nih.gov/, PRJNA1157790.

## Abbreviations

AR, androgen receptor; ACSL4, acyl-CoA synthetase long chain family member 4; BSA, bovine serum albumin; CH, Cepharanthine hydrochloride; DHODH, dihydroorotate dehydrogenase; DFO, deferoxamine; ECL, enhanced chemiluminescence; FSP1, ferroptosis suppressor protein 1; FTL, ferritin light chain; FTH1, ferritin heavy chain 1; Fer-1, ferrostatin-1; FBS, fetal bovine serum; GPX4, glutathione peroxidase 4; GA, gallic acid; HMOX1, heme oxygenase 1; IREB2, iron response element binding protein 2; MTT, methylthiazolyldiphenyl-tetrazolium bromide; PEI, polyethylenimine; PCa, prostate cancer; PEI, polyethylenimine; ROS, reactive oxygen species; SAT1, spermidine/spermine N1-acetyltransferase 1; TFRC, transferrin receptor; TEM, transmission electron microscope.
